# New Insights From MRI and Cell Biology Into the Acute Vascular-Metabolic Implications of Electronic Cigarette Vaping

**DOI:** 10.3389/fphys.2020.00492

**Published:** 2020-05-21

**Authors:** Felix W. Wehrli, Alessandra Caporale, Michael C. Langham, Shampa Chatterjee

**Affiliations:** ^1^Laboratory for Structural Physiologic and Functional Imaging, Department of Radiology, University of Pennsylvania, Philadelphia, PA, United States; ^2^Department of Physiology and Institute for Environmental Medicine, University of Pennsylvania, Philadelphia, PA, United States

**Keywords:** E-cigarette, endothelium, MRI, vaping, vascular

## Abstract

The popularity of electronic cigarettes (e-cigs) has grown at a startling rate since their introduction to the United States market in 2007, with sales expected to outpace tobacco products within a decade. Spurring this trend has been the notion that e-cigs are a safer alternative to tobacco-based cigarettes. However, the long-term health impacts of e-cigs are not yet known. Quantitative magnetic resonance imaging (MRI) approaches, developed in the authors’ laboratory, provide conclusive evidence of acute deleterious effects of e-cig aerosol inhalation in the absence of nicotine in tobacco-naïve subjects. Among the pathophysiologic effects observed are transient impairment of endothelial function, vascular reactivity, and oxygen metabolism. The culprits of this response are currently not fully understood but are likely due to an immune reaction caused by the aerosol containing thermal breakdown products of the e-liquid, including radicals and organic aldehydes, with particle concentrations similar to those emitted by conventional cigarettes. The acute effects observed following a single vaping episode persist for 1–3 h before subsiding to baseline and are paralleled by build-up of biological markers. Sparse data exist on long-term effects of vaping, and it is likely that repeated regular exposure to e-cig aerosol during vaping will lead to chronic conditions since there would be no return to baseline conditions as in the case of an isolated vaping episode. This brief review aims to highlight the potential of pairing MRI, with its extraordinary sensitivity to structure, physiology and metabolism at the holistic level, with biologic investigations targeting serum and cellular markers of inflammation and oxidative stress. Such a multi-modal framework should allow interpretation of the impact of e-cigarette vaping on vascular health at the organ level in the context of the underlying biological alterations. Applications of this approach to the study of other lifestyle-initiated pathologies including hypertension, hypercholesterolemia, and metabolic syndrome are indicated.

## Introduction

The popularity of electronic cigarettes (e-cigs) has grown at a startling rate since their introduction to the United States market in 2007, with sales expected to outpace tobacco products within a decade. Spurring this trend has been the notion that e-cigs are a safer alternative to tobacco-based cigarettes. However, the long-term health impact of e-cigs are not yet known, and the existing research does not support such a conclusion (Kligerman et al., 2020). The most urgent concern is the huge rise in the use of e-cigs by adolescents, showing an increase of over 87% in 12th graders from 2017 to 2019 with a 2019 prevalence of 35.1% (16.1% in 8th graders) (Miech et al., 2019).

Even though it has been conjectured that e-cig use may be less deleterious to human health than tobacco smoking, or perhaps not harmful at all, this notion has been challenged by a number of reports that appeared during the past several years, in the form of studies *in vitro* (Schweitzer et al., 2015), in animal models (Lerner et al., 2015) and, importantly, involving human subjects (Vlachopoulos et al., 2016; Biondi-Zoccai et al., 2019).

Besides nicotine, which has long been known to cause endothelial dysfunction (EDF) (Neunteufl et al., 2002), e-cig users are exposed to a host of toxic compounds generated by thermal degradation of solvents (Bekki et al., 2014; Jensen et al., 2017), and possibly flavorings (Omaiye et al., 2019), along with metal contaminants and ultrafine metal particles ejected by the heating element (Williams et al., 2013, 2017; Olmedo et al., 2018). Recent findings from the authors’ laboratories indicate that a single vaping episode involving non-nicotinized e-liquid provoked an inflammatory immune response and oxidative stress along with reduced nitric oxide (NO) bioavailability (Chatterjee et al., 2019b). The latter also manifested in impaired peripheral vascular reactivity and endothelial function as determined by a battery of quantitative magnetic resonance imaging (MRI) metrics (Caporale et al., 2019). Almost coincidentally with the release of our findings, a surge in vaping-related lung illnesses and deaths had been reported, variably attributed to additives such as vitamin-E acetate in cannabis-based vaping liquids (Lewis et al., 2019).

The purpose of this brief review is to highlight some of the recent findings from imaging studies, with particular focus on magnetic resonance, and to elaborate on the potential of these methods to gain insight into the vascular-metabolic consequences of e-cig vaping, notably EDF. We further show that the transient imaging findings are paralleled by rapid formation of signaling molecules, both in serum and in pulmonary endothelial cells, in response to e-cig aerosol exposure. The vascular-metabolic effects of other risk-alleviating tobacco products, e.g., heat-not-burn cigarettes, are not discussed here but we refer the reader to the key paper by Biondi-Zoccai et al. (2019).

## Endothelial Dysfunction and Its Manifestations at Micro and Macro-Cellular Level

The vascular circulatory system is a network that integrates all organ systems via the transport of blood, nutrients, and pathogenic stimuli. Chemical or physical alterations (Herrmann and Lerman, 2001) within the vascular system play a major role in vasodilatation and vasoconstriction of blood vessels. The endothelial layer that lines these vessels functions as a mechanical barrier, regulating fluid movement through blood vessels, but also participates in regulation of vessel tone. This process is mediated via endothelial derived relaxing factor (Furchgott and Zawadzki, 1980) later recognized to be NO, a product of endothelial cells. The endothelium is by virtue of its location, the converging site of inflammation toward which immune cells are recruited and into which these cells later adhere and extravasate. Indeed, as natural barrier to inflammatory moieties and microorganisms (from invading tissues) in the blood, the endothelium is also integral to innate immune activation in response to microbial attack. These events are facilitated by endothelial signaling that leads to production of adhesion molecules, cytokines and chemokines that drive immune cell adherence, inflammation, and endothelial oxidative stress. A major participant in endothelial signaling is reactive oxygen species (ROS). Endothelial cells produce ROS in response to stimuli ranging from physical (shear stress) to chemical (inflammatory agents, microbes, and smoke, etc.). ROS in turn activate signaling cascades that induce inflammation and oxidative stress leading to EDF, which entails compromised vasodilatory capacity. Vasorelaxation is expected, for example, in response to increased shear stress or pharmacologic stimuli (e.g., acetylcholine), but can be impaired as a result of reduced synthesis or depressed bioavailability of NO, the most potent endogenous vasodilator promoting activation of proinflammatory and prothrombic pathways, which in turn cause reduced blood fluidity (Bleakley et al., 2015). EDF is often found with pre-clinical inflammatory or autoimmune diseases [e.g., rheumatoid arthritis (Kerekes et al., 2008), diabetes mellitus (Tabit et al., 2010)], and promoted by hypertension (Bleakley et al., 2015) and unhealthy lifestyle (e.g., tobacco abuse, lack of physical activity, and improper diet) leading to hypercholesterolemia (Defago et al., 2014), as well as environmental factors such as air pollution (Bourdrel et al., 2017).

**FIGURE 1:**
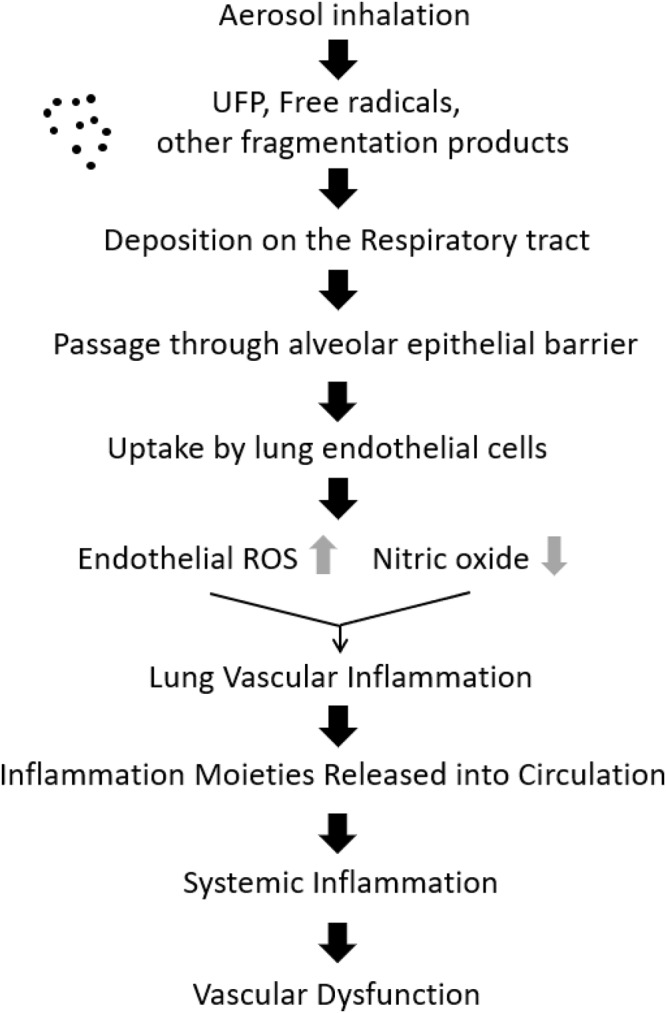
Cascade of events following e-cigarette inhalation, initiating vascular inflammation and dysfunction. Ultra-fine particles (UFP) and free radicals, deposited on the respiratory tract are taken up by endothelial cells. This process drives endothelial activation that triggers inflammation signaling, eventually leading to vascular dysfunction (original figure).

Of all the organs in the body, the lung is possibly the most vascularized. The pulmonary system is also in direct contact with the environment and for gas exchange to occur at the air-water interface, inhaled gases or particles must pass through alveolar surfactant, alveolar epithelium, and basement membrane into the capillary endothelium. In the case of inhaled smoke or particulate matter such as fine or ultra-fine particles associated with tobacco or e-cigs, the pulmonary endothelium responds to this exposure by activation of the endothelial enzyme NADPH oxidase to produce ROS (Chatterjee et al., 2019a). Pulmonary endothelial ROS produce intercellular adhesion molecule (ICAM) which is key to recruitment and adherence of immune cells from the circulation. ROS also react with NO, to form the highly reactive peroxynitrite that curtails NO bioavailability. As the production of peroxynitrite is accompanied by reduction in NO, it is also linked to EDF (Cannon, 1998). The cascade of events following e-cig aerosol inhalation is illustrated in Figure 1.

In addition to these changes occurring at the cellular level of the endothelium, inflammatory moieties that increase in the bloodstream in response to smoking or exposure to particulate matter, entail increased circulatory burden. In the long term, such insults can amplify inflammation along the endothelial layer by enhanced expression of cell surface adhesion molecules, enabling docking of circulating polymorphonuclear neutrophils to endothelial cells and their transmigration in the interstitium and airspace. It can also lead to changes between interconnected endothelial cells via increasing gap junctions (Doerschuk et al., 1999) making it more permissive to the spread of inflammation over large areas of the lung and compromising vascular properties such as vasodilation or vasoconstriction.

Thus, evaluation of changes at the microcellular level are crucial in gaining insights into events as they occur sequentially post inhalation: from the initial signals at the endothelial cellular level, to its macro-manifestations on the vascular network changes.

## Role of MRI as an Investigational Tool of E-Cigarette Induced Vascular and Metabolic Effects in Humans

The literature on image-based assessment of the effects of e-cigarettes on the cardiovascular system is sparse. Recent work using B-mode ultrasound has provided some insight into the acute effects of e-cigarette exposure in smokers in the form of flow-mediated dilation (*FMD*). A group of Italian investigators found brachial artery *FMD* in e-cigarette users to be impaired following a single vaping episode (Carnevale et al., 2016; Biondi-Zoccai et al., 2019).

Flow-mediated dilation is induced by transient increase in conduit blood flow velocity due to reduced microvascular resistance as the organism’s mechanism to re-oxygenate hypoxic tissue. The resulting increase in shear stress to the endothelium leads to vasodilation of the conduit artery via NO release (Widlansky et al., 2003). While often regarded as an effective surrogate marker for endothelial function (or dysfunction), the method’s poor intra-and inter-session reproducibility plague brachial artery *FMD* (Hardie et al., 1997; Ghiadoni et al., 2012).

Carotid-femoral pulse-wave velocity (*PWV*) has also been explored as a tool to study the acute impact of e-cigarette vaping (Vlachopoulos et al., 2016; Franzen et al., 2018). Both studies provided evidence of transient arterial wall stiffening following vaping and tobacco smoking. *PWV* is typically obtained by measuring the time delay of the systolic pressure wave at some downstream location, using pressure transducers placed at two locations (typically carotid and common femoral arteries) (Asmar et al., 1995; Wilkinson et al., 1998) or Doppler ultrasound (Sutton-Tyrrell et al., 2001).

Magnetic resonance imaging (MRI) is unmatched in its ability to provide structural information at high resolution and contrast, as well as quantitative physiologic and functional information non-invasively. It has provided pivotal insight into the vascular system in the form of quantitative perfusion data in the brain (Detre et al., 2009) and musculoskeletal system (Wu et al., 2009), blood flow velocity and flow rate of major arteries and veins (Markl et al., 2003) and, more recently, the metabolic rate of oxygen consumption, MRO_2_ (Xu et al., 2009; Jain et al., 2010), or parameters assessing vascular compliance (Mohiaddin et al., 1989) and *PWV* (Grotenhuis et al., 2009; Langham et al., 2011). MRI quantification of each of these physiologic quantities is able to yield results expressed in absolute physiologic units. MRI thus provides us with a sensitive toolbox to study vascular-metabolic disturbances such as acute and chronic effects of smoking and vaping, the topic of this review. Thus, unlike competing techniques, which evaluate a single physiologic property, MRI is able to measure a host of parameters covering multiple vascular territories in a single session, as first demonstrated in a study of the acute effects of tobacco smoking (Langham et al., 2015). In comparison to non-smokers, smokers had reduced endothelial function and vascular reactivity independent of subjects’ age. In that work, both measures of post-occlusion hyperemia and femoral *FMD* were assessed with an upper leg cuff-occlusion protocol disrupting both arterial inflow and venous return, along with parameters describing the time-course of femoral vein oxygen saturation (SvO_2_). The latter also yielded significant group differences, interpreted in terms of compromised microvascular reactivity (Langham et al., 2015).

**FIGURE 2:**
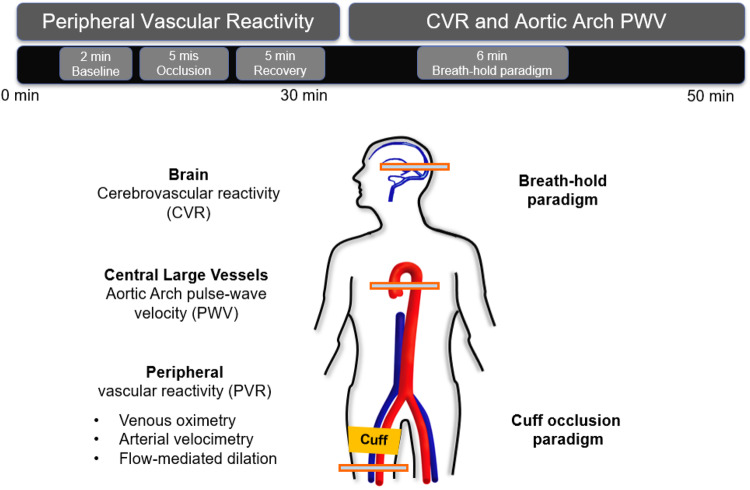
Multi-vascular MRI protocol. The 50-min MRI protocol included a measure of peripheral vascular reactivity (PVR) to cuff-induced ischemia, followed by cerebrovascular reactivity (CVR) and aortic pulse wave velocity (PWV) quantification. PVR was assessed in the femoral vessels via multiple techniques. To stimulate reactive hyperemia the cuff was placed around the upper thigh and inflated for 5 min. CVR to hypercapnia in the form of volitional apnea was measured in the superior sagittal sinus (artwork modified from Caporale et al., 2019; Supplementary Figure B1, with permission from RSNA publisher).

**FIGURE 3:**
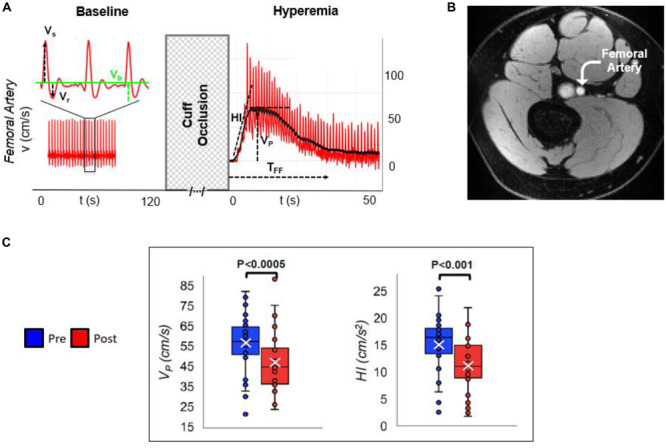
Arterial velocimetry at baseline and hyperemia. **(A)** Blood flow velocity in the superficial femoral artery, during baseline and post-cuff occlusion (reactive hyperemia). Systolic, retrograde, and baseline velocities are indicated (V_s_, V_r_, and V_b_, respectively). Post-ischemia, hyperemia is evaluated in terms of hyperemic index (HI) as the slope of the initial part of the velocity-time curve, peak average velocity (V_P_), and duration of hyperemia, referred to as time of forward flow (T_FF_). **(B)** Axial MR image of the thigh perpendicular to the femoral artery. **(C)** Pre- vs. post-electronic cigarette (e-cig) vaping differences for two parameters, in non-smokers, after a single episode of non-nicotinized e-cig vaping. The same MRI protocol was executed before and after e-cig use (modified from Caporale et al., 2019; Figures 2, 5, with permission from RSNA publisher).

**FIGURE 4:**
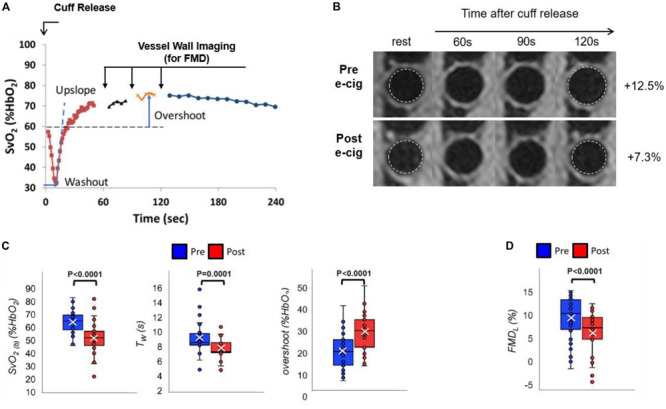
Venous oximetry and flow-mediated dilation. **(A)** Post-cuff release venous oxygen saturation (SvO_2_) interleaved with vessel wall imaging to derive femoral artery flow-mediated dilation (FMD) measurement at 60, 90, and 120 s following cuff deflation, with respect to the lumen at rest **(B)**. **(B–D)** Pre vs. post-e-cig vaping differences for the extracted metrics: **(B,D)** luminal FMD (FMD_L_), **(C)** pre-cuff occlusion baseline SvO_2_ [SvO_2(b)_], washout time (T_W_), and overshoot (**A**,**B** are original, produced from data already published in Caporale et al., 2019; **(C,D)** are modified from Caporale et al., 2019; Figure 5, with permission from RSNA publisher).

The above multi-vascular MRI procedure, which has since been refined, permits concurrent measurement of arterial and venous post-ischemia reactivity and endothelial function (Caporale et al., 2019), central aortic PWV (Langham et al., 2011) as well as cerebrovascular reactivity (Wu et al., 2019), to study acute effects of e-cigarette vaping (Figure 2; Caporale et al., 2019). The enhanced efficiency allows running the entire protocol in back-to-back sessions separated by a vaping episode. The objective of the latter study was to evaluate the acute transient effects of inhalation of nicotine-free e-cig aerosol in smoking-naïve young subjects (Caporale et al., 2019). The work was motivated by prior data indicating aerosol and its break-down products to contain toxic compounds that can enter the vascular system through upper respiratory pathways (Bakand et al., 2012; Nakane, 2012). Excerpts of the protocol, along with pre/post comparison data are shown in Figures 3, 4. The principle, along with sample data, for quantification of metrics of hyperemia of the femoral artery is illustrated in Figure 3, together with a comparison of two of the extracted parameters before and after vaping (Figures 3A,C, respectively). Figure 4A displays the femoral vein oxygen saturation curve following cuff release, interleaving this measurement with high-resolution femoral artery vessel wall images (Figure 4B) to derive FMD at three time-points. Pre/post plots of the oximetric parameter derived from the SvO_2_ recovery curve are presented in Figure 4C, the change in FMD in Figure 4D. All data are highly significant and consistent with the notion that the aerosol (supplemented with tobacco flavor), or the breakdown products (Wang et al., 2017), possibly micro-particles emanating from the heating elements (Williams et al., 2013, 2017), cause an immune response [as shown by the authors’ data in Chatterjee et al. (2019b)], leading to acute EDF.

## Relationship of Imaging Findings to Biological Markers

Endothelial dysfunction, a process occurring at the microvascular level, is highly predictive of future macrovascular events (Bleakley et al., 2015). Microvascular dysfunction manifests in alterations of endothelial receptors, and reduction in NO availability due to reduced biosynthesis and increased scavenging by oxygen radicals linked to oxidative stress. One consequence is impaired anti-inflammatory protection, leading to increased systemic inflammation, represented, for instance, by C-reactive protein (CRP) (Bleakley et al., 2015). Associations between subclinical vascular disease established by means of MRI and other quantitative imaging modalities, and vascular inflammation is well established [see, for instance (McEvoy et al., 2015) as well as some of the current authors’ prior work (Langham et al., 2015)]. Arterial FMD, as pointed out earlier, is mediated by NO release in the endothelium (Kooijman et al., 2008). The substantial reduction in femoral artery FMD measured with MRI in non-smokers approximately 20–30 min after inhalation of nicotine-free-cig aerosol (Caporale et al., 2019) is paralleled by a decrease in NO and increase in CRP in the circulating serum, recovering to baseline values within 2–3 h (Chatterjee et al., 2019b). Similarly, a decrease on the order of 20–30% in NO bioavailability was found in non-smokers, whose brachial artery FMD was reduced by almost 40% after exposure to nicotinized e-cigarette in two crossover studies comparing the effects of e-cig vaping to tobacco smoking (Carnevale et al., 2016; Biondi-Zoccai et al., 2019), where tobacco smoking was found to cause a larger decrement in both quantities). The above two studies further provided evidence of increased oxidative stress expressed by augmented NADPH oxidase activation, and increased 8-iso prostaglandin F2α, and reduced levels of vitamin E, an anti-oxidant, following exposure to both, e-cig aerosol and tobacco smoke (Carnevale et al., 2016; Biondi-Zoccai et al., 2019).

## Discussion and Conclusion

The data reviewed above compellingly show that quantitative MRI is extraordinarily sensitive to detect single events, in this case a vaping episode of nicotine-free aerosol. These results, though limited, demonstrate adverse effects consistent with transient impairment of endothelial function, along with reduced micro- and macrovascular reactivity. The time window for the observed effects is in the order of 1–3 h, which begs the question whether repeated vaping on this time scale causes a chronic response without the vascular system allowed to return to baseline conditions. Another question is which, among the myriad possible sources of toxins, would be the key culprit eliciting the observed responses. The studies reviewed in section “Role of MRI as an Investigational Tool of E-Cigarette Induced Vascular and Metabolic Effects in Humans” involved healthy, smoking naïve, young subjects rather than smokers/vapers. The latter would require study of both, acute and chronic exposure, which is currently in progress in the authors’ laboratory. The other unknown is the added effect of nicotine that, alone, can promote EDF.

## Author Contributions

FW, AC, and SC conceived and drafted the manuscript. FW, SC, AC, and ML read, edited, and approved the final manuscript.

## Conflict of Interest

The authors declare that the research was conducted in the absence of any commercial or financial relationships that could be construed as a potential conflict of interest.
